# Metabolic, Mitochondrial, and Inflammatory Effects of Efavirenz, Emtricitabine, and Tenofovir Disoproxil Fumarate in Asymptomatic Antiretroviral-Naïve People with HIV

**DOI:** 10.3390/ijms25158418

**Published:** 2024-08-01

**Authors:** Sergio Barroso, Mariona Guitart-Mampel, Francesc Josep García-García, Judith Cantó-Santos, Laura Valls-Roca, Félix Andújar-Sánchez, Adrià Vilaseca-Capel, Ester Tobías, Angela Arias-Dimas, Tania Quesada-López, Rafael Artuch, Francesc Villarroya, Marta Giralt, Esteban Martínez, Ester Lozano, Glòria Garrabou

**Affiliations:** 1 Inherited Metabolic Diseases and Muscular Disorders Research Lab, Cellex-Institut d’Investigacions Biomèdiques August Pi i Sunyer (IDIBAPS), Faculty of Medicine and Health Sciences, University of Barcelona (UB), 08036 Barcelona, Spain; sbarroro30@alumnes.ub.edu (S.B.); mguitart@recerca.clinic.cat (M.G.-M.); fjgarcia@recerca.clinic.cat (F.J.G.-G.); jcanto@recerca.clinic.cat (J.C.-S.); lvalls@recerca.clinic.cat (L.V.-R.); fandujsa7@alumnes.ub.edu (F.A.-S.); avilasca9@alumnes.ub.edu (A.V.-C.); etobiasb@ub.edu (E.T.); 2 Department of Internal Medicine, Hospital Clinic of Barcelona, 08036 Barcelona, Spain; 3 CIBERER-Spanish Biomedical Research Centre in Rare Diseases, Carlos III Health Institute, 28029 Madrid, Spain; rafael.artuch@sjd.es; 4 Department of Clinical Biochemistry, Institut de Recerca Sant Joan de Déu, Esplugues de Llobregat, 08950 Barcelona, Spain; angelayasmina.arias@sjd.es; 5 Biochemistry and Molecular Biomedicine Department, Biomedicine Institute (IBUB), University of Barcelona (UB), 08014 Barcelona, Spain; tquesada@ub.edu (T.Q.-L.); fvillarroya@ub.edu (F.V.); mgiralt@ub.edu (M.G.); 6 CIBER Physiopathology of Obesity and Nutrition (CIBEROBN), Carlos III Health Institute, 28029 Madrid, Spain; 7 Infectious Diseases Department, Hospital Clinic of Barcelona, 08036 Barcelona, Spain; estebanmartinez@ub.edu; 8 CIBER of Infectious Diseases (CIBERINFEC), Carlos III Health Institute, 28029 Madrid, Spain; 9 Department of Cell Biology, Physiology and Immunology, Faculty of Biology, University of Barcelona (UB), 08028 Barcelona, Spain

**Keywords:** antiretroviral treatment, HIV, metabolic profile, mitochondrial toxicity, mitochondrial DNA, inflammatory effects, tenofovir, emtricitabine, efavirenz

## Abstract

This study aimed to comprehensively assess the metabolic, mitochondrial, and inflammatory effects of first-line efavirenz, emtricitabine, and tenofovir disoproxil fumarate (EFV/FTC/TDF) single-tablet regimen (STR) relative to untreated asymptomatic HIV infection. To this end, we analyzed 29 people with HIV (PWH) treated for at least one year with this regimen vs. 33 antiretroviral-naïve PWH. Excellent therapeutic activity was accompanied by significant alterations in metabolic parameters. The treatment group showed increased plasmatic levels of glucose, total cholesterol and its fractions (LDL and HDL), triglycerides, and hepatic enzymes (GGT, ALP); conversely, bilirubin levels (total and indirect fraction) decreased in the treated cohort. Mitochondrial performance was preserved overall and treatment administration even promoted the recovery of mitochondrial DNA (mtDNA) content depleted by the virus, although this was not accompanied by the recovery in some of their encoded proteins (since cytochrome c oxidase II was significantly decreased). Inflammatory profile (TNFα, IL-6), ameliorated after treatment in accordance with viral reduction and the recovery of TNFα levels correlated to mtDNA cell restoration. Thus, although this regimen causes subclinical metabolic alterations, its antiviral and anti-inflammatory properties may be associated with partial improvement in mitochondrial function.

## 1. Introduction

The human immunodeficiency virus (HIV) is responsible for the development of acquired immunodeficiency syndrome (AIDS), but it is also known to produce diffuse metabolic, mitochondrial, and inflammatory damage in people with HIV (PWH) who have not developed AIDS [1,2]. Over the past decades, combined Antiretroviral Therapy (cART) has significantly reduced the morbidity and mortality associated with HIV-infection becoming a lifelong therapy [3,4]. The latest studies have shown the importance of early treatment initiation to also reduce the risk of both AIDS and non-AIDS-related events [5,6]. Additionally, the benefits of early treatment also include decreasing infection rates [7]. The combination of different antiretroviral drugs is crucial for the effective management of HIV infection and the prevention of drug resistance [8]. Nonetheless, some families of antiretroviral drugs have also been associated with a myriad of secondary effects [9].

First-designed nucleoside-analogue reverse transcriptase inhibitors (NRTIs), such as zidovudine and stavudine, are well-known mitochondrial toxins due to off-target impairment of mitochondrial DNA (mtDNA) ɣ-polymerase, with consequent blockage of mitochondrial genome replication and associated mitochondrial and metabolic dysfunction [10,11]. This inhibition can lead to bioenergetic deficits, oxidative stress, and apoptosis, clinically associated with secondary effects, including peripheral neuropathy, myopathy, lactic acidosis, lipodystrophy syndrome, or hyperlactatemia [12,13,14,15]. On the other hand, last-generation NRTIs, Tenofovir Disoproxil Fumarate (TDF) and Emtricitabine (FTC), have been demonstrated to have a safer mitochondrial toxic profile. However, they are not exempt from a certain degree of toxicity [16,17]. Other antiretroviral families, such as protease inhibitors (PIs), have been linked to molecular and clinical toxicity, particularly metabolic changes and premature aging. They interact with cellular proteins, such as the cellular retinoic acid binding protein 1, the low-density lipoprotein receptor-related protein 1, and the GLUT1/GLUT4 glucose transporters inducing mitochondrial toxicity through oxidative stress, leading to an indirect disruption of the overall function of the mitochondrial respiratory chain (MRC). These effects contribute to conditions like metabolic syndrome, lipodystrophy, hepatic steatosis, and insulin resistance [18,19,20]. The non-nucleoside-analogues transcriptase inhibitors (NNRTIs) can also induce moderate mitochondrial damage through disruptions of the mitochondrial membrane potential [21]. Indeed, some NNRTIs, such as Efavirenz (EFV) and Nevirapine, have been related to apoptosis and direct MRC dysfunction; thus, producing a subsequent fall in ATP production [22]. This alteration may increase oxidative stress which may trigger apoptotic events, promoting a pro-inflammatory state [14]. Consequently, lipid vacuoles tend to be stored in hepatic cells, producing a hepatic steatosis like that observed in the NRTI family of antiretroviral drugs [23,24]. This range of secondary effects prompted the design of safer alternatives for drug families, such as integrase strand transfer inhibitors (INSTIs), CCR5 antagonists, and HIV fusion blockers.

Historically, cART regimens consisted of two NRTIs combined with a PI, but the drug families like INSTIs, are now preferred for initial treatment due to their efficacy and minimal mitochondrial impact [25,26,27]. For instance, the European AIDS Clinical Society Guidelines 2023 recommend a first-line regimen with a high barrier to resistance, including a combination of NRTIs plus a second-generation INSTI [28]. In this sense, INSTIs have shown a generally favorable mitochondrial safety profile, although minor mitochondrial toxicity has not been entirely ruled out [29,30].

Over the years, the toxicity of cART regimens has been significantly reduced, and their therapeutic administration simplified. Currently, the majority of PWH take a single-tablet regimen (STR) [31]. These regimens offer the convenience of oral administration and simplified dosing. However, the previously described secondary effects of these new formulations have been scarcely explored and some of them, such as lipodystrophy, can persist even while using new antiretroviral families [32,33]. The first combination for cART in a STR included two NRTIs (TDF plus FTC), along with one NNRTI, EFV [34,35]. This schedule provided a simplified treatment option for PWH and was the worldwide gold standard formulation until 2015, being still the standard-of-care cART in some developing countries [36,37]. This STR has been shown to be a good alternative to first-line schedules, especially where INSTIs are contraindicated, but also when concomitant tuberculosis infection is present [28]. This combination exemplifies the NNRTI-based STR still in use today, offering effective viral suppression with a manageable safety profile. For instance, this drug combination has also been indicated for HIV patients with reported dyslipidemia, since switching from other regimens to TDF/FTC/EFV not only maintains virologic response but also significantly improves key lipid parameters [14]. However, some concerns have been raised on the hepatic toxicity of this combination and the potential development of lactic acidosis, a surrogate marker of metabolic and mitochondrial toxicity [38], promoting the assessment of molecular and toxic effects in treated patients.

Alongside the constant development of novel agents to fight HIV infection, there is also a need to enhance the methodological approaches to assess their concomitant noxious effects with low invasiveness. In this sense, it is typical to evaluate the metabolic effects of antiretroviral interventions, but few studies incorporate mitochondrial toxicity (mitox), inflammatory markers of drug effects, or include the assessment of drug toxicity by using high-throughput and easily exportable surrogate biomarkers [27,28]. For instance, the assessment of mitox encompassing the measurement of mtDNA or its content in peripheral blood mononuclear cells (PBMCs–mtDNA) is essential to evaluate the real impact of toxicity on mitochondrial performance [39,40]. In this context, new plasma biomarkers of mitox are being proposed to reduce invasiveness, such as the plasma cell-free mtDNA (plasma-mtDNA) [41,42]. In this study, we also incorporated plasma biomarkers traditionally used to study and diagnose metabolic and mitochondrial disorders, such as fibroblast growth factor 21 (FGF21), also linked to metabolic syndrome [43,44,45], and coenzyme Q10 (CoQ), both potential surrogates of MRC dysfunction [46]. The interplay between induced mitox and inflammation involves a complex network of soluble factors. We assess the relationship between antiretroviral induced for its treatment and other inflammatory soluble mediators, such as hepatocyte growth factor (HGF) [47], nerve growth factor (NGF) [48], interleukin 6 (IL-6) [49,50], IL-8 [51], serum leptin [52], monocyte chemoattractant protein 1 (MCP-1/CCL2), and tumor necrosis factor α (TNFα) [53]. These cytokines participate in immune response and tissue repair processes; thus, their involvement in the context of mitox can influence cellular metabolism, oxidative stress, and inflammatory pathways [41].

Considering the different side effects that can be attributed to cART, it is crucial to evaluate the potential clinical benefits, but also the potential noxious effects of STR in cohort studies. This analysis may be especially important in asymptomatic individuals with preserved CD4 cell counts where the individual impact of antiretroviral therapy may not be immediately evident. Hence, we compared asymptomatic PWH with no comorbidities in naïve condition vs. PWH treated for at least one year of TDF/FTC/EFV. We aim to show a comprehensive and complete fingerprint of drug toxicity at different levels (metabolic, mitochondrial, and inflammatory response) by using novel and standard biomarkers (ideally easy-exportable).

## 2. Results

### 2.1. Clinical and Hematological Characteristics of HIV-1 Infected Patients Comparing Naïve vs. Treated with cART

A total of 62 PWH with no comorbidities were included in this study, of whom 33 were naïve and 29 were on antiretroviral treatment. Table 1 summarizes the clinical characteristics of both cohorts. There were no significant differences in gender distribution between both groups (chi-square statistic = 0.799, *p*-value = 0.372). Age and time since diagnosis were higher in the treated group since this study included 17 patients before and after treatment (Supplementary Table S1 summarizes the longitudinal analysis results for paired data within this group before and after cART showing similar results to those of cross-sectional analysis). As expected, this combination regimen significantly decreased viral load while recovering CD4^+^ T cell count. In addition, mean cell volume (MCV), mean corpuscular hemoglobin (MCH), and platelet count were also significantly higher in treated patients compared to naïve HIV-infected patients (Table 1).

### 2.2. Metabolic Parameters of HIV-1 Infected Patients Comparing Naïve vs. Treated with cART

**Figure 1:**
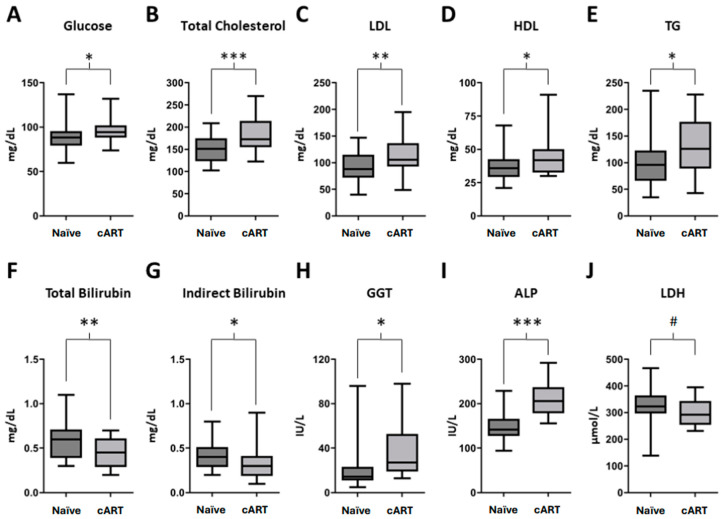
Metabolic parameters in Naïve HIV patients (n = 33) compared to patients treated with cART (combined antiretroviral therapy) based on TDF/FTC/EFV for more than one year (n = 29). (**A**) Plasma glucose values; (**B**) Total Cholesterol values; (**C**) LDL: LDL-Cholesterol values; (**D**) HDL: HDL-Cholesterol values; (**E**) TG: Triglycerides levels; (**F**) Total bilirubin levels; (**G**) Indirect bilirubin levels; (**H**) GGT: Gamma-glutamyl transferase values; (**I**) ALP: Alkaline phosphatase values; (**J**) LDH: Lactate Dehydrogenase values. Box and whiskers plots showing median, minimum, and maximum values. # *p*-value = (0.05–0.1), * *p*-value < 0.05, ** *p*-value < 0.01, *** *p*-value < 0.001.

We next wanted to assess metabolic alterations by evaluating standard biochemical parameters in both cohorts. As shown in Figure 1, we found a significant elevation of metabolic parameters in patients treated with cART regarding plasma glucose (*p* = 0.010; Figure 1A), as well as in several markers of the lipidic profile, such as total cholesterol (*p* < 0.001; Figure 1B), Low-Density Lipoprotein cholesterol (LDL); *p* = 0.004; Figure 1C), High-Density Lipoprotein cholesterol (HDL; *p* = 0.018; Figure 1D), and triglycerides (TG; *p* = 0.048; Figure 1E). Contrarily, when comparing total cholesterol vs. HDL ratio differences, results did not reach statistical significance (Supplementary Table S2).

Additionally, cART-treated patients showed lower values of total and indirect bilirubin but increased levels of hepatic enzymes gamma-glutamyl transferase (GGT) and alkaline phosphatase (ALP) (Figure 1F–I), suggesting a plausible reduction in hemolysis processes while inducing subclinical levels of drug-induced hepatotoxicity. Although not statistically significant, there was a decrease in the lactate dehydrogenase (LDH) plasmatic values (*p* = 0.066; Figure 1J) and uric acid levels (*p* = 0.090; Supplementary Table S2). Supplementary Table S2 summarizes additional results of metabolic parameters for both cohorts, including apolipoproteins A1 and B, creatine kinase, total proteins, amylase and lipase, sodium, potassium and renal parameters.

To sum up, the significant increment in glucose levels and all lipid profile values, together with alterations in parameters of the hepatic function, suggest a subclinical metabolic toxicity produced by this STR.

### 2.3. Mitochondrial Parameters of HIV-1 Infected Patients Comparing Naïve vs. Treated with cART

**Figure 2:**
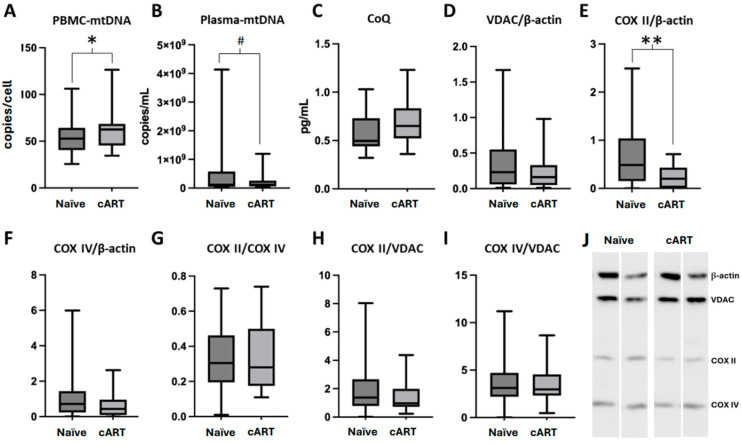
Mitochondrial parameters in Naïve HIV patients (n = 33) compared to patients treated with cART (combined antiretroviral therapy) based on TDF/FTC/EFV for more than one year (n = 29). (**A**) PBMC-mtDNA: Peripheral blood mononuclear cells mitochondrial DNA; (**B**) Plasma-mtDNA: Plasma mitochondrial DNA levels; (**C**) CoQ: Coenzyme Q values; (**D**); VDAC: Voltage-Dependent Anion-selective Channel vs. β-actin ratio; (**E**) COX-II: Cytochrome c oxidase subunit II vs. β-actin ratio; (**F**) COX IV: Cytochrome c oxidase subunit IV vs. β-actin ratio; (**G**) COX-II/COX-IV ratio; (**H**) COX-II/VDAC ratio; (**I**) COX-IV/VDAC ratio. Box and whiskers plots showing median, minimum, and maximum values. # *p*-value = (0.05–0.1), * *p*-value < 0.05, ** *p*-value < 0.01. (**J**) Representative Western Blot bands for proteins quantification in PBMC from two Naïve HIV patients compared to two patients treated with TDF/EFV/EFV STR for more than one year: β-actin (47 kDa), VDAC (31 kDa), COX-II (25.6 kDa) and COX-VI (15 kDa).

To assess the mitox caused by this therapeutic regimen, we measured the levels of mtDNA in PBMCs and plasma as well as the protein levels of key components of the mitochondrial respiratory chain. As shown in Figure 2, we found a significant increase in the PBMCs–mtDNA from patients treated with the cART TDF/FTV/EFV STR for more than one year (*p* = 0.045; Figure 2A), suggesting the cell recovery of mtDNA previously depleted by the virus. Interestingly, plasma-mtDNA was lower in treated patients (Figure 2B), although results did not reach statistical significance (*p* = 0.074), confirming that cell mtDNA leakage into the plasma had also been decreased on account of parallel HIV reduction. Interestingly, protein levels of the cytochrome c oxidase subunit II (COX-II) subunit normalized to β-actin were significantly decreased with this therapeutic regimen (*p* = 0.005; Figure 2E,J) and no significant differences were found in either CoQ, or the other studied mitochondrial subunits (Figure 2C,F–I). Therefore, despite the increase in mtDNA promoted by antiretroviral treatment in PBMCs is not being associated with a recovery in the protein levels of COX-II yet, the rest of mitochondrial parameters were preserved, suggesting minimal effects on mitochondrial performance.

### 2.4. Inflammatory and Soluble Mediators of HIV-1 Infected Patients Compared with Naïve vs. Treated with cART

**Figure 3:**
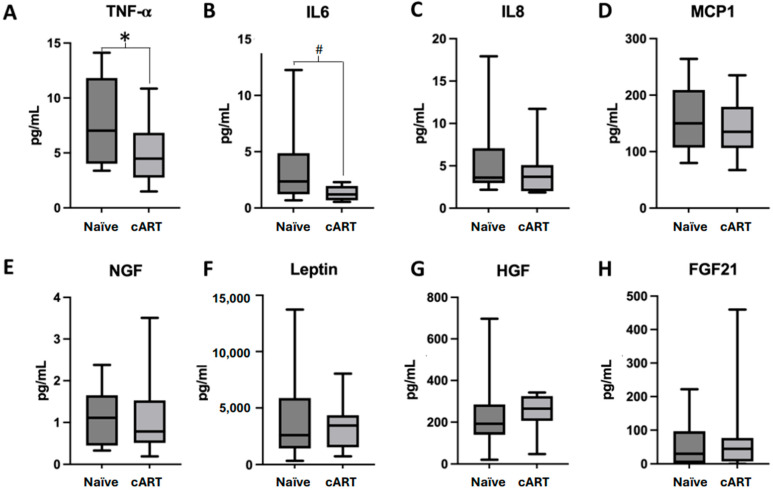
Inflammatory and soluble mediators in Naïve HIV patients (n = 15) compared to patients treated with cART (combined antiretroviral therapy) based on TDF/FTC/EFV for more than one year (n = 10). (**A**) TNFα: Tumor Necrosis Factor α levels. (**B**) IL6: Interleukin 6 values; (**C**) IL8: Interleukin 8 values; (**D**) MCP-1: Monocyte Chemoattractant protein 1 levels; (**E**) NGF: Nerve Growth Factor values; (**F**) Leptin: Serum Leptin levels; (**G**) HGF: Hepatocyte Growth Factor levels; (**H**) FGF21: Fibroblast Growth Factor 21 levels. Box and whiskers plots showing median, minimum, and maximum values. # *p*-value = (0.05–0.1) * *p*-value < 0.05.

To investigate the levels of subclinical inflammation caused by this cART combination, we next quantified the concentration of proinflammatory cytokines and soluble growth factors in the plasma of both cohorts. As shown in Figure 3, proinflammatory cytokine TNFα was significantly decreased after TDF/FTC/EFV treatment (*p* = 0.043; Figure 3A), in accordance with viral suppression. Consistently, IL-6 (and to a lesser extent IL-8 and MCP-1) was reduced in treated patients although it did not reach statistical significance (*p* = 0.068; Figure 3B–D). No significant differences were found in the other parameters studied (Figure 3E–H), confirming the safety and positive effect of this antiretroviral schedule at the inflammatory level.

### 2.5. Significant Correlations between Virologic and Metabolic Parameters with Mitochondrial Biomarkers in Both Cohorts

**Figure 4:**
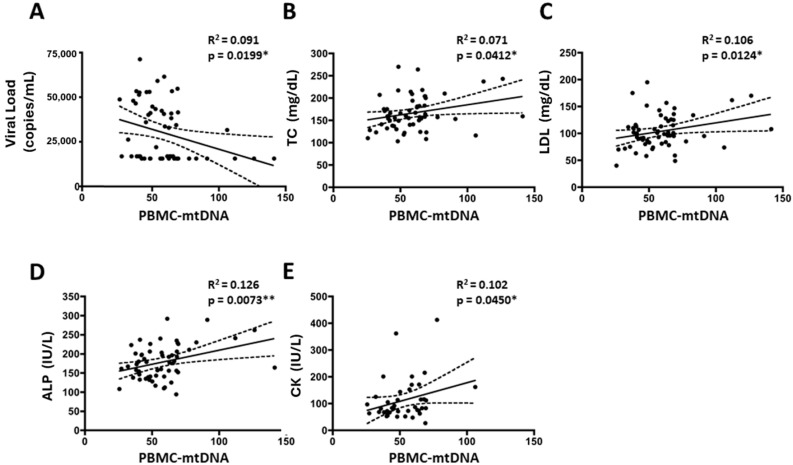
Significant correlations between virologic and metabolic parameters with mitochondrial biomarkers in both cohorts. Linear regression line (solid line) and 95% confidence band of the best-fit line (dotted line) are shown. (**A**) Correlation between mitochondrial DNA content in PBMCs (PBMC-mtDNA) vs. patient viral load; (**B**) Correlation between PBMC-mtDNA vs. total cholesterol values; (**C**) Correlation between PBMC-mtDNA vs. LDL values; (**D**) Correlation between PBMC-mtDNA vs. alkaline phosphatase (ALP) values; (**E**) Correlation between PBMC-mtDNA vs. Creatine Kinase values. * *p*-value < 0.05, ** *p*-value < 0.01.

We next explored the molecular associations between the infectious and metabolic parameters studied in these cohorts with markers of mitochondrial function. Among the studied mitochondrial parameters, PBMC-mtDNA content was found to be significantly elevated in treated patients and inversely correlated with patient viral load (Figure 4A). On the contrary, this biomarker of mitochondrial function positively correlated with altered metabolic parameters, such as total cholesterol, LDL, ALP, and creatine kinase (Figure 4B–E), suggesting that cART treatment worsens metabolic profile while lessening the viral injury; thus, allowing the recovery of PBMC-mtDNA content. Consequently, PBMC-mtDNA represents a useful biomarker to detect mitochondrial alterations and is associated with several metabolic alterations found in treated patients.

### 2.6. Significant Correlations between Virologic and Mitochondrial Parameters with Inflammatory Biomarkers in Both Cohorts

**Figure 5:**
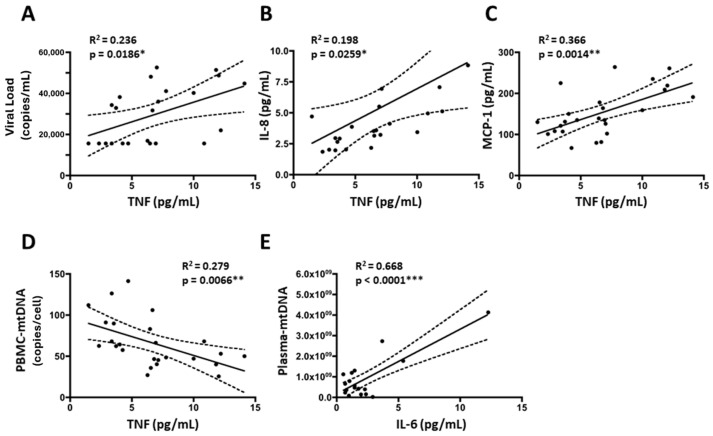
Significant correlations between virologic and mitochondrial parameters with inflammatory biomarkers in both cohorts. Linear regression line (solid line) and 95% confidence band of the best-fit line (dotted line) are shown. (**A**) Correlation between TNFα vs. viral load; (**B**) Correlation between Tumor Necrosis Factor α (TNFα) vs. IL-8 values; (**C**) Correlation between TNFα vs. Monocyte Chemoattractant protein 1 (MCP-1) values; (**D**) Correlation between TNFα vs. mitochondrial DNA in PBMCs; (**E**) Correlation between IL-6 vs. mitochondrial DNA in plasma. * *p*-value < 0.05, ** *p*-value < 0.01, *** *p*-value < 0.0001.

In addition, to explore the molecular associations between studied parameters, we investigated whether levels of the proinflammatory cytokine TNFα (that were reduced in treated patients), could be associated with changes in mitochondrial biomarkers. In accordance with HIV-mediated inflammation, levels of TNFα positively correlated with viral load (Figure 5A). As expected, levels of the proinflammatory cytokine TNFα positively correlated with levels of chemokines IL-8 (CXCL8) and MCP-1 (CCL2) (Figure 5B,C). Interestingly, mtDNA levels within cells (PBMC-mtDNA) were reduced by the presence of the virus and have typically been altered by previously used cART mitox schedules when both agents exert mitochondrial toxicity. Conversely, plasma-mtDNA levels were increased by the cell lysis and mtDNA leakage into the bloodstream promoted by the virus. Consequently, since the present cART treatment reduced viral load without causing mitochondrial toxicity, we observed negative correlations between inflammation (TNFα) and cell mtDNA (PBMC-mtDNA, Figure 5D) and positive correlations between inflammation (proinflammatory cytokine IL-6) and plasma-mtDNA (Figure 5E). Taken together, our data indicate that subclinical inflammation and mtDNA recovery are associated, probably because both improve due to HIV-decrease on account of cART administration. However, the increase in PBMC-mtDNA did not successfully translate into the complete recovery of mitochondrial function since some MRC subunits encoded by mtDNA (such as COX-II) remained reduced in cART-treated patients. Therefore, mitochondrial performance was preserved overall and inflammation was ameliorated by antiretroviral administration.

## 3. Discussion

Despite the well-established therapeutic efficacy of cART using FDA-approved antiretroviral drugs, these medications are often linked to a wide range of metabolic alterations and mitox, leading to significant adverse effects, such as lipodystrophy, metabolic syndrome, and hepatic or renal impairment [14,54]. Moreover, TDF/FTC/EFV is still the most frequently used combination in some countries, like Thailand and many resource-limited settings, according to the WHO guidelines [37] and it is being tested alone and in combination with inhibitors of the HIV viral protein U to improve modulation of inflammatory and immune function [55]. However, and despite this regimen has been used throughout the years, scarce knowledge is available on its metabolic, mitochondrial, and inflammatory profile that may eventually lead to the manifestation of further adverse clinical effects, especially when administered in STR.

In our study, the administration of the TDF/FTC/EFV drug regimen reached the main goal of reducing viral load to undetectable levels while increasing CD4^+^ T cell count. These findings confirmed the excellent therapeutic activity of the TDF/FTC/EFV treatment, which is also supported by previous studies [34,35,36]. However, the analysis of metabolic parameters showed some alterations in the glucidic and lipidic profiles, such as increased levels of plasma glucose, total cholesterol, LDL, HDL, and TG. Part of these metabolic alterations had been published previously [56,57], demonstrating an increased lipid profile specifically caused by TDF/FTC/EFV compared to other combinations, such as TDF/FTC/Rilpivirine [58] or TDF/Doravirine/Lamivudine [59]. Consistent with our results, previous studies showed a decrease in LDH as a parameter for HIV treatment monitoring [60]. In our cohort, cART-treated patients also showed lower values of total and indirect bilirubin but increased levels of hepatic enzymes GGT and ALP, suggesting subclinical drug-induced hepatotoxicity, probably due to an intrahepatic cholestasis mechanism, as previously suggested [61,62,63]. This data is also in line with other reports showing similar metabolic alterations for new-generation schedules [25,64,65]. Additionally, the increase of ALP may also be explained by an increase in bone metabolism caused by the TDF included in this formulation [57]. On the other hand, the decrease in total and indirect bilirubin could be attributed to hemolysis processes occurring during HIV infection, resulting in the release of indirect bilirubin levels that are improved after treatment [58]. The reduction in uric acid levels, although not statistically significant, is supported by previous studies where this metabolite is proposed as an antioxidant [66]. Additionally, this decrease may also indicate improved renal function after cART [67]. Likewise, when analyzing patient hematological characteristics, there was an increase in MCV, MCH, and platelet account, suggesting a plausible maturation disturbance in the erythropoiesis process, supported by others [59] that was reversed by the treatment. A better understanding of the molecular mechanisms involved in metabolic cART toxicity will improve the development of novel therapeutic strategies.

Mitox is one of the main concerns in cART for PWH since mitochondria impairment plays a significant role in the pathogenesis of a wide range of disorders, such as peripheral neuropathy, myopathy, lactic acidosis, lipodystrophy syndrome, or hyperlactatemia [12,13,14,18,68]. Previous studies reported that the downregulation of signature genes controlling mitochondrial biogenesis and metabolism in CD4^+^ T cells derived from PWH on antiretroviral treatment (tenofovir-based regimens) supported mitochondrial dysfunction [69]. In this sense, the mitochondrial toxic effects of antiretrovirals, such as the decrease in the mtDNA ɣ-polymerase activity, translate into a decrease in mtDNA, followed by a downstream reduction of mitochondrial-encoded proteins [70]. The decrease in key proteins participating in mitochondrial homeostasis affects the normal energetic function. In addition, these effects are enhanced by mitochondrial membrane potential disruptions and increased oxidative stress, leading in some cases to proapoptotic and proinflammatory states [21]. Long-term clinical manifestations of mitox range from hyperglycemia and hyperlipidemia to insulin resistance, hepatic toxicity, and steatosis [14,18,23,24]. These alterations, frequently associated with metabolic syndrome and premature aging, highlight the importance of monitoring mitochondrial health in HIV treatment. In this study, we found that mitochondrial performance was preserved overall and treatment administration even promoted the recovery of mtDNA content depleted by the virus. Recovery of mtDNA is the first step to restoring mitochondrial function, correlating with enhanced biogenesis, respiratory chain function, and reduced oxidative stress; thus, mitigating associated clinical adverse effects of mitox [71,72,73,74]. However, in our study, mtDNA recovery was not accompanied by the restoration in some of their encoded proteins (since COX-II significantly decreased). Whether their levels may eventually be recovered after long-term TDF/FTC/EFV treatment (over one year herein tested) is still a matter of doubt. In any case, mitox profiling was safe, after treatment administration.

The inflammatory profile also ameliorated after treatment in accordance with viral reduction exemplified by TNFα and IL-6 reductions. Indeed, the concentration of the proinflammatory cytokine TNFα was significantly lower in treated patients and positively correlated with viral load. This infers increased inflammatory processes in patients living with higher viral load rates. On the other hand, other inflammatory molecules showed no significant differences in plasmatic levels; thus, confirming the safety profile of this schedule. Taken together, cART-treated patients showed decreased TNFα and recovery of the PBMC-mtDNA content indicating that this regimen may reduce subclinical inflammation and partially improve mitochondrial function. Recovery of mtDNA is a well-documented indicator of improved mitochondrial function, correlating with enhanced biogenesis, respiratory chain function, and reduced oxidative stress [71,72,73,74]. In this regard, we previously associated mitochondrial dysfunction and inflammation in other clinical settings, such as patients with sepsis, where both mitox and inflammation correlated with disease severity and outcome [75].

As previously mentioned, one of the strengths of this study is the combination of classical and new biomarkers for toxicity profiling, widening the scope of molecular targets and sensibility scores of methodological approaches. Importantly, PBMC-mtDNA levels were an excellent biomarker to assess cART toxicity since it was correlated with clinical parameters associated with infection (viral load), metabolic alterations (total cholesterol, LDL, ALP, creatin kinase), and inflammation (TNFα). Another strength is that we were able to display differences in metabolic, mitochondrial, and inflammatory parameters due to the presence of the virus in untreated naïve patients which allowed us to differentiate between the effects of the treatment and the effects of the virus itself. We confirmed viral mitochondrial toxicity with depleted PBMC-mtDNA content in association with increased concentrations of proinflammatory levels caused by the virus itself. We also demonstrated the toxicity of TDF/FTC/EFV schedule at the subclinical metabolic level. Ideally, it would have been of interest to include a third cohort of non-infected patients on antiretroviral therapy to assess the effects of the treatment without viral interference, but this is not feasible in clinical settings. In this regard, we previously reported that uninfected patients who received HIV post-exposure prophylaxis (PEP) showed subclinical mitochondrial damage after 28 days of treatment [76], but the effects after one year (or more) of specific TDF/FTC/EFV therapy had not been tested before without viral interference. Likewise, the observed increases in lipid concentrations may be influenced by a return-to-health response, particularly relevant for individuals initiating cART after prolonged untreated HIV infection [77,78]. However, since the mean of CD4 count in the Naïve group was within reference values, minor return-to-health effect in this group would be expected, that may or not be linked to lipid increases. On the other hand, we must acknowledge the limited sample size of our cohorts that may be hindering statistical significance. In addition, we acknowledge that metabolic and mitox parameters may be more exacerbated in high energy-dependent tissues, such as liver or muscle [79]. However, the recruitment of these samples would require invasive approaches that struggle with ethical considerations in the clinical management of PWH.

In conclusion, treatment of more than one year of first-line TDF/FTC/EFV STR maintains an excellent therapeutic activity while preserving or improving mitochondrial and inflammatory profiles [34,35,36]. However, subclinical metabolic alterations emerge considering lipid, glucidic, and hepatic profiles, that should be taken into consideration, especially in the case of patients of advanced age or signs of metabolic syndrome. Despite these alterations remain subclinical in the present study, long-term treatment may lead to the manifestation of clinical adverse effects of these metabolic disarrangements. Further metabolic, mitochondrial, and inflammatory studies should be conducted to monitor these parameters in asymptomatic patients to anticipate subclinical alterations and prevent drug-induced toxicity.

## 4. Materials and Methods

### 4.1. Patient Cohorts

A single-site observational and cross-sectional study was conducted comparing two different populations of PWH with different control and treatment options A total of 62 patients with no comorbidities were included: 29 asymptomatic PWH who had received a first-line therapy consisting of 12 to 24-months of antiretroviral treatment with TDF/FTC/EFV (cART cohort) vs. 33 asymptomatic and PWH who have never been administered any antiretroviral or any other drugs (naïve cohort). Of them, 17 were longitudinal studied patients (before and after intervention), but the rest were cross-sectionally included patients to increase sample size. TDF/FTC/EFV (tenofovir disoproxil fumarate 245 mg, emtricitabine 200 mg, and efavirenz 600 mg), was provided under the STR—Atripla^®^ (Bristol-Myers Squibb and Gilead Sciences Limited, IDA Business & Technology Park, Carrigtohill, County Cork, Ireland), as it was the gold-standard regimen until 2015. Among the 33 naïve patients, 17 were eventually treated with the treatment with TDF/FTC/EFV for more than a year and were also included in the cART cohort. All patients gave written informed consent to participate in the study, which was approved by the Ethics Committee of the Hospital Clinic of Barcelona (Register code: 2010/5865) (08036, Barcelona, Spain). Epidemiological, immunological, and therapeutic parameters including age, sex, years since HIV-1 diagnosis, and months of cART exposure were recorded.

### 4.2. Sample Collection

After an overnight fast, 20 mL of venous blood were collected in Vacutainer^TM^ EDTA tubes (BD Biosciences, Franklin Lakes, NJ, USA). Blood was first centrifuged at room temperature for 15 min at 1500 g and plasma was stored at −80 °C until use. PBMCs were isolated by Ficoll density gradient centrifugation (Histopaque^®^-1077, Sigma Diagnostics, St. Louis, MO, USA). After isolation, an aliquot of 20 million of PBMCs were resuspended in 100 μL of phosphate-buffered saline (PBS) and stored frozen at −80 °C to obtain a cell-lysate for analysis.

### 4.3. Infection Markers

The lymphocyte subpopulation count (CD4 and CD8) was performed using a FACSCanto^TM^ II cytometer and data were analyzed with FlowJo software v.10.9 (BD Biosciences, Franklin Lakes, NJ, USA). Plasma HIV-1 RNA levels were determined through quantitative PCR using the COBAS 6800 system (Roche Diagnostics, Mannheim, Germany), with a quantification limit of 37 copies/mL.

### 4.4. Metabolic Toxicity Markers

A complete blood count was conducted. Metabolic parameters were determined through molecular absorption spectrophotometry using the Siemens Atellica Solution CH (Siemens Healthineers, Erlangen, Germany). This system was used to measure glucose, total cholesterol, HDL, LDL, TG, AST, ALT, GGT, creatin kinase, LDH, and total proteins. Apolipoprotein A1 and Apolipoprotein B were measured by immunoturbidimetry on the Atellica Solution CM analyzer (Siemens Healthineers, Erlangen, Germany).

### 4.5. Total DNA Isolation from PBMCs and mtDNA Quantification

An aliquot of 10 μL including 2 million of PBMCs was used to prepare a cell-lysate for extracting total DNA by a standard phenol-chloroform procedure. To evaluate mtDNA content, fragments of the mitochondrially encoded 12S rRNA gene and the nuclear-encoded RNase-P gene were amplified separately by quantitative PCR using TaqMan^®^ Gene Expression Master Mix (Applied Biosystems, Waltham, MA, USA), in a total amount of 25 ng of extracted DNA. To determine mitochondrial mtDNA, MtF805 (5′-CCACGGGAAACAGCAGTGAT-3′) was used as the mt12S rRNA forward primer and MtR927 (5′-CTATTGACTTGGGTTAATCGTGTGA-3′) was used as the mt12S rRNA reverse primer, using a TaqMan probe 6FAM-5′-TGCCAGCCACCGCG-3′-MGB, from Applied Biosystems. As an endogenous reference for nuclear DNA, a commercial kit was used (RNase P Control Reagent VIC, part no 4316844; Applied Biosystems). We used an internal calibration curve for mitochondrial and nuclear DNA quantification, and final mtDNA content was expressed as the ratio between mitochondrial and nuclear DNA amount (ratio mt12SrRNA vs. RNase-P) [68].

### 4.6. Nucleic Acid Isolation from Plasma and Quantification of mtDNA

DNA was extracted from 200 μL of plasma using QIAmp DNA Minikit (QIAGEN, Alameda, CA, USA). Each DNA sample was eluted in 50 μL of supplied buffer and stored at −20 °C until use. Mitochondrial DNA content in plasma was analyzed by rt-PCR, as previously described [63].

### 4.7. Mitochondrial Protein Quantification from PBMCs by Western Blot

Western blot immunoassay was performed in 20ug of cell lysate to assess COX-II (encoded by mtDNA) and cytochrome c oxidase subunit IV (COX-IV) subunit (encoded by nuclear DNA). COX-II and COX-IV quantification was normalized by the voltage-dependent anion-selective channel (VDAC; mitochondrial-encoded protein) and β-actin (nuclear-encoded protein), respectively, to establish the relative mitochondrial protein expression as we previously reported [12].

### 4.8. Enzyme-Linked Immunosorbent Assay (ELISA) and Multiplex Assays

The quantification of FGF21 levels were measured using a human specific ELISA (Biovendor, Brno, Czech Republic). HGF, IL-6, IL-8, leptin, MCP-1, NGF, and TNFα levels were determined using a Multiplex system (Lynco Research/Merck, Darmstadt, Germany) and a Luminex 100ISv2 instrument (Thermo Fisher Scientific, Waltham, MA, USA), using plasma dilutions recommended by each commercial kit.

### 4.9. Coenzyme Q10 (CoQ) Quantification

Plasma CoQ was measured by reverse-phase high-performance liquid chromatography with electrochemical as reported elsewhere [80].

### 4.10. Statistical Analysis

After filtering for outlier detection, differences between groups were determined with the two-tailed T test or Mann-Whitney U test based on normality results. Correlation between quantitative parameters was assessed by using the Pearson or Spearman correlation test as indicated. Non-parametric statistical analysis for paired data was performed using the Wilcoxon test. Statistical significance was defined as *p*-value < 0.05, but border-line significances ranging 0.05 to 0.1 have been also highlighted along the article. Statistical tests were performed using the Statistical Package for Social Sciences version 25.0 (SPSS, Chicago, IL, USA).

## Figures and Tables

**Table 1:** Clinical and hematological characteristics of HIV-1 infected patients Naïve (n = 33) vs. patients after TDF/FTC/EFV treatment (n = 29).

	Naïve	TDF/FTC/EFV	*p*-Value
Sex (male n, %)	33 (28, 84.8%)	29 (22, 75.9%)	0.372
Age (years)	37.24 ± 1.7	40.83 ± 1.8	0.051
Time since diagnosis (years)	3.27 ± 0.5	5.2 ± 0.7	0.019 *
Time on cART (months)	-	22.3 ± 2.6	-
CD4 count (cells/mm^3^)	473.06 ± 30.2	610.14 ± 36.7	0.003 **
Log (Viral Load)	4.38 ± 0.2	1.60 ± 0.01	<0.001 ***
White blood cells (×10^9^/L)	5.89 ± 0.3	6.51 ± 0.4	0.321
Hemoglobin (g/L)	143.9 ± 2.4	147.0 ± 2.0	0.335
Hematocrit (%)	43.0 ± 0.6	44.2 ± 0.6	0.200
MCV (fL)	88.7 ± 0.6	92.9 ± 0.6	<0.001 ***
MCH (pg)	29.7 ± 0.3	31.0 ± 0.2	0.001 **
MCHC (g/L)	334.5 ± 1.9	332.5 ± 2.0	0.529
RDW (%)	13.4 ± 0.1	13.1 ± 0.2	0.097
HDW (g/L)	26.8 ± 0.5	25.9 ± 0.4	0.197
Hypochromic (%)	1.36 ± 0.4	0.84 ± 0.2	0.285
Platelets (×10^9^/L)	215.84 ± 9.1	263.4 ± 11.4	0.004 **
MPV (fL)	8.3 ± 0.1	8.6 ± 0.2	0.292

Data are presented as Mean ± SEM. * *p*-value < 0.05, ** *p*-value < 0.01, *** *p*-value < 0.001. Abbreviations: TDF, Tenofovir Disoproxil Fumarate; FTC, Emtricitabine; EFV, Efavirenz; MCV, mean cell volume; MCH, mean corpuscular hemoglobin; MCHC, mean corpuscular hemoglobin concentration; RDW, red cell distribution width; HDW, hemoglobin distribution width; MPV, Mean platelet volume.

## Data Availability

The data presented in this study are available on request from the corresponding authors. The data are not publicly available due to ethical restrictions and will be shared in accordance with consent provided by participants on the use of confidential data.

## Supplementary Materials

The following supporting information can be downloaded at: https://www.mdpi.com/article/10.3390/ijms25158418/s1.
