# Supplementary material for: Metabolic, Mitochondrial, and Inflammatory Effects of Efavirenz, Emtricitabine, and Tenofovir Disoproxil Fumarate in Asymptomatic Antiretroviral-Naïve People with HIV
Source: Int J Mol Sci. 2024 Aug 1;25(15):8418. doi: 10.3390/ijms25158418 (PMC11313075; doi:10.3390/ijms25158418)
Supplement: Supplementary file 1 [file ijms-25-08418-s001.zip › ijms-3049129-supplementary.pdf]

Supplementary Table S1: Paired data analysis for patients before and after more than one year of TDF/FTC/EFV treatment. (n=17). Wilcoxon test results for paired data analysis are indicated. Despite safety of mitochondrial and inflammatory profile, subclinical metabolic alterations were observed for study patients after more than one year of treatment, independently if we considered only those with longitudinal follow-up (n=17, in this table) or between study cohorts (n=33 vs. 29, in the rest of the article).

PARAMETERS	NAÏVE (Mean ± SEM)	TDF/FTC/EFV	p-value
**Age**	38.65 ± 2.3	40.5 ± 2.1	0.001*
**TREATMENT OUTCOMES**			
**CD4 count**	460 ± 42	626 ± 37	0.013*
**LogVL**	4.8 ± 0.30	1.61 ± 0.01	<0.0001*
**HEMOGRAM**			
**Leucocytes**	5.9 ± 0.5	7.0 ± 0.5	0.022*
**Hemoglobin**	144 ± 3.3	149 ± 2.5	0.096#
**Hematocrit**	43.1 ± 0.9	44.9 ± 0.9	0.019*
**MCV**	88.9 ± 1.0	94.0 ± 0.6	<0.0001*
**MCH**	29.9 ± 0.3	31.2 ± 0.3	0.001*
**MCHC**	335.6 ± 2.0	331.5 ± 2.5	0.068#
**RDW**	13.4 ± 0.2	13.2 ± 0.2	0.300
**HDW**	27.0 ± 0.6	25.5 ± 0.5	0.024*
**Hypochromic**	1.06 ± 0.2	0.77 ± 0.2	0.232
**Platelets**	213 ± 9	267 ± 14	0.002*
**MPV**	8.3 ± 0.2	8.6 ± 0.3	0.331
**METABOLIC PARAMETERS**			
**Carbohydrate and lipid metabolism**			
**Glucose**	90.35 ± 3.8	98.29 ± 3.1	0.039*
**Total Cholesterol**	154.06 ± 7.0	176.71 ± 8.5	0.044*
**LDL-Cholesterol**	95.13 ± 6.6	106.76 ± 6.6	0.088#
**HDL-Cholesterol**	38.06 ± 2.6	45.7 ± 3.5	0.015*
**TG**	108.24 ± 13.2	122.06 ± 12.6	0.381
**Apolipoprotein A1**	110.14 ± 5.8	125.7 ± 13.5	0.180
**Apolipoprotein B**	76.86 ± 9.1	96.50 ± 4.9	0.655
**FGF21**	61.98 ± 16.8	65.51 ± 31.2	0.460
**Leptin**	6506 ± 3632	4748 ± 1923	0.593
**Hepatic function**			
**AST**	27.12 ± 2.6	23.65 ± 1.6	0.367
**ALT**	30.71 ± 5.0	24.65 ± 3.1	0.609
**Total Bilirubin**	0.51± 0.03	0.43 ± 0.04	0.046*
**Direct Bilirubin**	0.16 ± 0.03	0.16 ± 0.02	0.655
**Indirect Bilirubin**	0.36 ± 0.04	0.26 ± 0.03	0.041*
**GGT**	31.29 ± 9.5	40.53 ± 10.2	0.024*
**ALP**	165.38 ± 7.3	209.0 ± 8.1	0.003*
**HGF**	274 ± 83	288 ± 24	1.000
**Catabolic parameters**			
**LDH**	321.69 ± 15.2	297.92 ± 13.7	0.255
**CK**	88.67 ± 15.2	119.54 ± 26.8	0.109
**Total Proteins**	77.35 ± 1.6	73.92 ± 1.3	0.161
**Pancreatic function**			
**Amylase**	63.20 ± 4.7	69.77 ± 8.6	0.262
**Lipase**	36.4 ± 3.4	33.83 ± 2.8	0.283
**Renal function**			

**Na**	140.24 ± 0.4	140.31 ± 0.5	0.855
**K**	4.40 ± 0.1	4.42 ± 0.1	0.688
**Creatinine**	0.94 ± 0.02	0.89 ± 0.03	0.029*
**GFR**	>60 ± 0.0	>60 ± 0.0	1.000
**Uric Acid**	5.36 ± 0.2	4.71 ± 0.3	0.028*
**INFLAMMATION**			
**IL6**	4.68 ± 2.2	1.67 ± 0.6	0.180
**IL8**	5.33 ± 1.6	3.1 ± 0.7	0.273
**MCP-1**	151.48 ± 29.8	135.59 ± 36.2	0.715
**NGF**	1.34 ± 0.5	2.00 ± 0.8	0.285
**TNFα**	9.8 ± 1.9	5.99 ± 1.8	0.144
**MITOCHONDRIAL PARAMETERS**			
**PBMC-mtDNA**	51.94 ± 3.2	57.79 ± 3.4	0.326
**Plasma-mtDNA**	7.4E8 ± 0.1E8	1.8E8 ± 0.5E8	0.112
**CoQ**	Not analyzed	Not analyzed	Not analyzed
**VDAC/β-actin**	0.40 ± 0.10	0.29 ± 0.10	0.388
**COXII/β-actin**	0.83 ± 0.16	0.23 ± 0.06	0.041*
**COXIV/β-actin**	1.00 ± 0.29	0.49 ± 0.15	0.328
**COXII/COXIV**	0.31 ± 0.07	0.31 ± 0.06	1.000
**COXII/VDAC**	2.45 ± 0.71	1.58 ± 0.41	0.272
**COXIV/VDAC**	2.79 ± 0.44	2.68 ± 0.53	0.975

Data are presented as Mean ± SEM. * p-value < 0.05, # p-value (0.1-0.05). Abbreviations: TDF, Tenofovir Disoproxil Fumarate; FTC, Emtricitabine; EFV, Efavirenz; CD4 count [cells/μL]; LogVL: Log viral load [log copies/mL]; Leucocytes [$\times 10^9$/L]; Hemoglobin [g/L]; Hematocrit [%]; MCV: Mean Cell Volume [fL]; MCH: Mean Corpuscular Hemoglobin [pg]; MCHC: Mean Corpuscular Hemoglobin Concentration [g/L]; RDW: Red cell Distribution Width [%]; HDW: Hemoglobin Distribution Width [g/L]; Hypochromic [%]; Platelets [$\times 10^9$/L]; MPV: Mean Platelet Volume [fL]; Glucose [mg/dL]; Total Cholesterol [mg/dL]; LDL-Cholesterol [mg/dL]; HDL-Cholesterol [mg/dL]; TG: Triglycerides [mg/dL]; Apolipoprotein A1 [mg/dL]; Apolipoprotein B [mg/dL]; FGF21: Fibroblast Growth Factor 21 [pg/mL]; Leptin [pg/mL]; AST: Aspartate aminotransferase [IU/L]; ALT: Alanine aminotransferase [IU/L]; Total Bilirubin [mg/dL]; Direct Bilirubin [mg/dL]; Indirect Bilirubin [mg/dL]; GGT: Gamma-glutamyl transferase [IU/L]; ALP: Alkaline phosphatase [IU/L]; HGF: Hepatocyte Growth Factor [pg/mL]; LDH: Lactate dehydrogenase [IU/L]; CK: Creatine Kinase [IU/L]; Total Proteins [g/L]; Amylase [IU/L]; Lipase [IU/L]; Na: Sodium [mEq/L]; K: Potassium [mEq/L]; Creatinine [mg/dL]; GFR: Glomerular Filtration Rate [mL/min]; Uric Acid [mg/dL]; IL6: Interleukin 6 [pg/mL]; IL8: Interleukin 8 [pg/mL]; MCP-1: Monocyte chemoattractant protein-1 [pg/mL]; NGF: Nerve Growth Factor [pg/mL]; TNFα: Tumor Necrosis Factor alpha [pg/mL]; PBMC-mtDNA: Mitochondrial DNA in peripheral blood mononuclear cells [copies/cell]; Plasma-mtDNA: Mitochondrial DNA in plasma [copies/mL]; CoQ: Coenzyme Q [pg/mL]; VDAC/β-actin: Voltage-dependent anion channel normalized to beta-actin; COXII/β-actin: Cytochrome c oxidase subunit II normalized to beta-actin; COXIV/β-actin: Cytochrome c oxidase subunit IV normalized to beta-actin; COXII/COXIV: Ratio of cytochrome c oxidase subunit II to subunit IV; COXII/VDAC: Ratio of cytochrome c oxidase subunit II to voltage-dependent anion channel; COXIV/VDAC: Ratio of cytochrome c oxidase subunit IV to voltage-dependent anion channel.

Supplementary Table S2: Additional metabolic parameters of study HIV- infected patients Naïve *vs.* patients after more than one year of TDF/FTC/EFV STR.

PARAMETERS	NAÏVE (n=33)	TDF/FTC/EFV (n=29)	p-value
**Carbohydrate and lipid metabolism**			
**TC/HDL ratio**	4.12 ± 0.1	4.38 ± 0.2	0.466
**ApoA1**	113.89 ± 6.8	125.67 ± 13.5	0.518
**ApoB**	79.56 ± 7.6	96.50 ± 4.9	0.104
**Hepatic function**			
**AST**	33.47 ± 6.1	27.04 ± 2.6	0.244
**ALT**	32.12 ± 5.0	28.11 ± 3.2	0.778
**Catabolism parameters**			
**Creatine Kinase**	107.62 ± 13.6	113.9 ± 23.5	0.860
**Total Protein**	77.36 ± 1.1	74.7 ± 1.3	0.155
**Pancreatic function**			
**Amylase**	72.64 ± 4.3	68.1 ± 7.1	0.560
**Lipase**	38.48 ± 2.4	33.6 ± 2.2	0.195
**Renal function**			
**Na^+**	140.43 ± 0.3	139.7 ± 0.6	0.245
**K^+**	4.39 ± 0.1	4.4 ± 0.1	0.831
**Creatinine**	0.93 ± 0.02	0.90 ± 0.02	0.213
**GFR**	>60 ± 0.0	>60 ± 0.0	1.000
**Uric Acid**	5.34 ± 0.2	4.8 ± 0.3	$0.090^{\#}$

Data are presented as Mean ± SEM. * p-value < 0.05, $^{\#}$ p-value (0.1-0.05). Abbreviations: TDF, Tenofovir Disoproxil Fumarate; FTC, Emtricitabine; EFV, Efavirenz; TC/HDL: Total Cholesterol vs. HDL ratio; ApoA1: Apolipoprotein A1 [mg/dL]; ApoB: Apolipoprotein B [mg/dL]; AST: Aspartate aminotransferase [IU/L]; ALT: Alanine aminotransferase [IU/L]; Creatine Kinase [IU/L]; Total Protein [g/L]; Amylase [IU/L]; Lipase [IU/L]; Na+: Sodium [mEq/L]; K+: Potassium [mEq/L]; Creatinine [mg/dL]; GFR: Glomerular Filtration Rate [mL/min]; Uric Acid [mg/dL].
